# Contact tracing during the COVID-19 outbreak: a protocol for enabling rapid learning from experiences and exploring the psychological impact on contact tracers

**DOI:** 10.12688/hrbopenres.13236.1

**Published:** 2021-03-30

**Authors:** Róisín O'Donovan, Claire Buckley, Philip Crowley, Hugh Fulham-McQuillan, Brynne Gilmore, Jennifer Martin, Eilish McAuliffe, Gemma Moore, Emma Nicholson, Éidín Ní Shé, Mary Clare O'Hara, Ricardo Segurado, Mary Rose Sweeney, Patrick Wall, Aoife De Brún

**Affiliations:** 1 UCD Centre for Interdisciplinary Research, Education, and Innovation in Health Systems (UCD IRIS), School of Nursing, Midwifery & Health Systems, University College Dublin, Dublin, Ireland; 2 Specialist in Public Health Medicine, Contact Management Programme, HSE and School of Public Health, University College Cork, Cork, Ireland; 3 National Quality Improvement Team, Health Service Executive, Dublin, Ireland; 4 School of Population Health, University of New South Wales, Sydney, Australia; 5 General Manager, HSE COVID-19 Contact Management Programme, Contact Tracing Centre, Galway, Ireland; 6 School of Public Health, Physiotherapy and Sports Science, University College Dublin, Dublin, Ireland; 7 School of Nursing, Psychotherapy and Community Health, Dublin City University, Dublin, Ireland

**Keywords:** Psychological impact, health services improvement, contact tracing, COVID-19, mixed methods research

## Abstract

**Background: **Given the unprecedented nature of the COVID-19 pandemic, the Irish health system required the redeployment of public sector staff and the recruitment of dedicated contact tracing staff in the effort to contain the spread of the virus. Contact tracing is crucial for effective disease control and is normally carried out by public health teams. Contact tracing staff are provided with rapid intensive training but are operating in a dynamic environment where processes and advice are adapting continuously. Real-time data is essential to inform strategy, coordinate interconnected processes, and respond to needs *.* Given that many contact tracers have been newly recruited or redeployed, they may not have significant experience in healthcare and may experience difficulties in managing the anxieties and emotional distress of the public.

**Aim:** (i) identify emerging needs and issues and feed this information back to the Health Service Executive for updates to the COVID-19 Contact Management Programme (CMP); (ii) understand the psychological impact on contact tracers and inform the development of appropriate supports.

**Methods: **We will use a mixed-methods approach. A brief online survey will be administered at up to three time points during 2021 to measure emotional exhaustion, anxiety, general health, and stress of contact tracing staff, identify tracing systems or processes issues, as well as issues of concern and confusion among the public. Interviews will also be conducted with a subset of participants to achieve a more in-depth understanding of these experiences. Observations may be conducted in contact tracing centres to document processes, practices, and explore any local contextual issues.

**Impact: **Regular briefs arising from this research with data, analysis, and recommendations will aim to support the work of the CMP to identify problems and implement solutions. We will deliver regular feedback on systems issues; challenges; and the psychological well-being of contact tracing staff.

## Introduction

Evidence suggests that approximately 80% of coronavirus disease 2019 (COVID-19) cases have mild symptoms, 14% of people infected become severely unwell, 6% become critically ill and the fatality rate, which increases with age, is influenced by other underlying conditions ^1^. Due to the disease’s highly communicable nature, the World Health Organization (WHO) describes contact tracing as one of the critical interventions for prevention and containment during outbreaks ^2^. Contact tracing involves contacting and providing advice to those who test positive for COVID-19 as well as those they have been in close contact with. Along with widespread testing and quarantine, contact tracing has been successful in reducing the incidence of COVID-19 and both COVID-19 specific and excess deaths ^3^. Optimising contact tracing processes and coverage in order to reduce the delay in testing individuals for COVID-19 is central to controlling the spread of the virus both during the initial stages of an epidemic as well as during de-escalation of physical distancing ^4, 5^. Silent incubation periods, unsuspected and undetected cases, and the speed of international spread through air travel make it particularly difficult to manage the spread of this infectious disease ^6^. Contact tracing, along with follow up quarantine and isolation measures, have been used to control transmission of other infectious diseases including severe acute respiratory syndrome (SARS) ^7^, Ebola ^8^, smallpox, tuberculosis, HIV, and syphilis ^9– 13^. Whilst there is a clear risk of acquiring COVID-19 from confirmed cases, another risk is posed by the late detection or delayed isolation of the likely cases ^8^. Poor contact tracing has been implicated in prolonging the duration of previous infectious disease outbreaks ^14^. While contact tracing is not new, the scope and nature of the COVID-19 pandemic means it has attracted renewed attention as the “linchpin” of epidemic control ^15^.

Given the unprecedented scale and impact of the COVID-19 pandemic, the Irish health system has required the redeployment of public sector staff and the recruitment of dedicated contact tracing staff in the efforts to contain the spread of the virus at various stages during the pandemic response. With confirmed cases spread across the country, the national health service has been working with partner organisations to establish contact tracing centres. As of February 2021, community-based contacts are identified and monitored by Health Service Executive (HSE) Departments of Public Health and the Contact Management Programme (CMP) while healthcare workers are identified and monitored by their organisations’ Occupational Medicine department, and hospital in-patient contacts are identified and monitored by infection prevention and control (IPC) and clinical microbiology ^16^. There are currently 788 staff working in 8 contact tracing centres (CTCs) across Ireland. A cloud-based system, named the Covidcare Tracker, has been developed to support contact tracing. A similar approach has been adopted in other national health systems across the EU ^17^.

There is considerable potential to harness the experience of contact tracers in the pivotal role they play by identifying needs and issues as they emerge in this rapidly changing situation. This includes challenges experienced during the contact tracing process as well as patterns in the public’s understanding of, and reported adherence to, the public health measures ^15^. Contact tracers are in an ideal position to escalate certain groups and/or complex cases to the relevant regional Departments of Public Health, such as people living in insecure and cramped accommodation or those who fear taking time off from work in order to isolate ^18, 19^. As a result, contact tracers play a crucial role in times of outbreaks and can provide useful insights that support and promote the effectiveness of tracing efforts ^20^. Previous research has used feedback from contact tracers to inform the development of strategies to adapt communication to promote contacts’ adherence ^6^. In this context, up to date data become essential for informing strategy, coordinating interconnected processes, troubleshooting problems, and responding to identified needs. Therefore, it is crucial that we mobilise learning from contact tracers for immediate impact and improvement of processes and systems.

It is important to acknowledge that contact tracing staff have varied backgrounds and may not necessarily have significant experience working in healthcare. While staff were provided rapid intensive training in contact tracing, including remote training sessions, live/in-person role play, practice sessions along with monitoring and coaching provided by more experienced tracers, they are performing challenging work and operating in a dynamic environment where processes and advice are adapting continuously, considering emerging evidence. The practice of contact tracing involves more than the ability to make a phone call; it requires the skills to impart clear and consistent public health advice in line with the current guidelines (and call scripts) in a manner that is empathic, reassuring and easily understandable, handle information, and collect and manage data ^15^. Contact tracing involves a set of varied roles and contextualised practices, such as communicating clearly, listening, decision making, negotiation and deliberation ^14^. Contact tracers may experience pressure in the role when required to make a high volume of calls, particularly during surges in cases and numbers of close contacts ^21^.

In addition, contact tracers are working in extraordinary and difficult times with recent studies showing higher levels of adverse psychiatric outcomes among the public since the beginning of the pandemic. A recent systematic review reported relatively high rates of symptoms of anxiety (6.33% to 50.9%), depression (14.6% to 48.3%), posttraumatic stress disorder (7% to 53.8%), psychological distress (34.43% to 38%), and stress (8.1% to 81.9%) among the general population during the COVID-19 pandemic in China, Spain, Italy, Iran, the US, Turkey, Nepal, and Denmark ^22^. In Ireland, evidence suggests that the pandemic has had a negative impact on well-being and mental health ^23^. In addition to coping with these challenges themselves, contact tracers may experience difficulties in managing the anxieties and emotional distress of people who may be severely ill and contacts of those that are unwell or recently deceased ^6^. They need to be able to build trust with the contacts they are calling and may need to provide support as they are delivering bad news ^24^.

While there is increasing recognition of the need for psychosocial support for frontline healthcare staff affected during epidemics or pandemics ^25^, relatively little attention has been paid to the potential emotional burden on contact tracers ^6^. Contact tracers receive rapid intensive training and are supported by more experienced tracers but some may lack previous experience of dealing with these sensitive issues. This has significant implications for the health and well-being of contact tracers themselves. While the contact tracing training currently offers self-care advice and national and local level supports including a psychological first aid service, research to understand the real-time impact of this work on staff is necessary to understand better how we can support individuals in this crucial role. 

### Research questions

RQ1. What are the challenges experienced (system/ process/ role/ practical issues) and information needs identified by contact tracers?

RQ2a. What is the psychological impact of contact tracing on staff during the COVID-19 pandemic response?

RQ2b. Does the psychological impact of contact tracing on staff during the COVID-19 pandemic response vary according to the demographic profile of tracers (i.e., training background and experience, gender, age)?

## Methods

### Study design and setting

**Figure 1.**
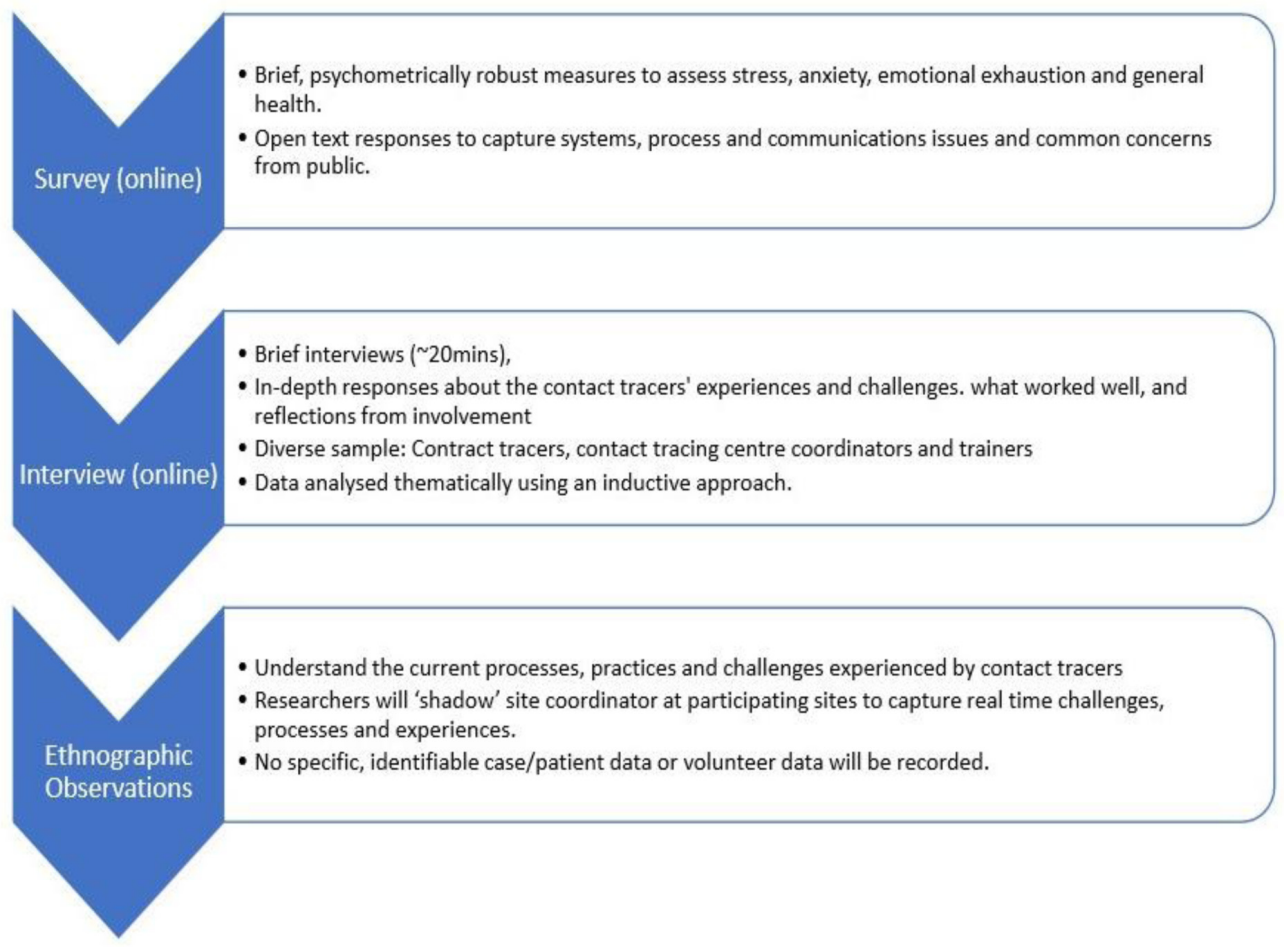
Overview of the study design.

This study will be conducted with contact tracing staff working in contact tracing centres (CTCs) across Ireland. We will use a mixed method approach to address the research questions and provide a rich understanding of contact tracers’ experiences during the outbreak. To address RQ1, we will conduct ethnographic observations (if public health guidance allows) and interviews to understand the current processes, practices and challenges experienced by contact tracers as the situation develops and as processes change and adapt to new learning. An online survey for contact tracers will be delivered to address RQ2. All participants will be invited to take part in both the survey and an interview, however, both aspects are on a volunteer basis and they are not required to take part in both. An overview of the study design can be found in Figure 1.

### Survey

Brief and psychometrically robust measures will be used to ask participants to report their level of stress, anxiety, emotional exhaustion, and general health ^26^. The survey will be hosted online via Qualtrics, which is GDPR (general data protection regulation) compliant. We propose to invite contact tracers to complete a brief survey at three points, approximately two months apart, during a 6-month data collection period. Participants will be invited to participate through the national health service via the CMP. Emails with information will be forwarded to the contact tracing centre site coordinators to disseminate among their staff. Individuals will then be invited to complete a brief online survey and/or to take part in an interview with a member of the research team. Informed consent will be obtained from participants at the start of the survey. Pseudo-anonymous identifiers will be used to track individual survey respondents over time.

The survey will include scales measuring emotional exhaustion (measured using the Maslach Burnout Inventory - General Survey (MBI-GS) using a 4 point scale ranging from “strongly agree” to “strongly disagree”) ^27^, perceived stress (measured using the Perceived Stress Scale using a 5 point scale ranging from “never” to “very often”) ^28^, general health (measured using the General Health Questionnaire, using a 4 point scale ranging from “much more than usual” to “much less than usual”) ^29^ state anxiety (measured using the Intrinsic Motivation Inventory, using a 7 point scale ranging from “not at all true” to “very true”) ^30^ and posttraumatic stress reactions (measured using the Impact of Event Scale-6 using 5 point scale ranging from “not at all” to “extremely”) ^31^. The survey will capture demographic details and will also include some open text boxes to capture information regarding specific/common issues that were raised during calls with cases/contacts and to identify any systems or process issues or challenges.

Independent and paired sample t-tests and mixed effects logistic regression will be used to explore changes over time. Power analyses were performed using G*Power ^32^ to estimate sample size requirements. Given a power level of .80, assuming a medium effect size of 0.5 and following a Bonferroni adjustment to account for multiple planned tests, the sample size required for an independent samples t-tests will be approximately 222, and the sample size necessary for a paired samples t-tests will be approximately 59. Provided that adequate sample sizes are achieved, subgroup analysis will be conducted to explore differences between respondents based on training background (i.e., healthcare training, counselling/ psychology training or other), age and gender.

### Interviews

Interviews will be conducted with contact tracers, CTC leads, trainers, redeployed staff and recruited contact tracing staff. We aim to recruit a diverse sample of contact tracers (up to 40 across three time points, or until thematic saturation has been reached), site coordinators and trainers (up to 12) across CTCs via a combination of purposive and snowball sampling to gather a range perspectives and experiences. These interviews will collect in-depth responses about the contact tracers’ experiences and challenges ^26^. The interview topic guide was developed in collaboration with members of the COVID-19 Contact Management Programme and includes open ended questions regarding participants experiences in their role, including training, ongoing support, experiences during contact tracing shifts. From site coordinators and trainers, we will gather experiences of implementation of the tracing programme, its evolution and adaptation, understanding challenges, what worked well, and reflections from involvement in the process. Our collaborators, the Health Service Executive National CMP and CTC coordinators, will support recruitment of contact tracers through dissemination of invitations to participate in interviews.

Interviews will be kept as brief as possible (~20mins) and will be conducted via an online platform (e.g., Zoom/Skype). Interviewees may participate at multiple time points and will be invited to follow-up interviews to explore changes in experiences over time and to ensure on-going learning from their experiences. With the participants’ informed consent, interviews will be audio-recorded, transcribed verbatim and analysed thematically using an inductive approach to identify key patterns and themes in the data ^33^. This analysis will be conducted using the data management programme NVivo 12 ^34^. We will analyse data on an on-going basis. Codes generated in the first round of data collection will inform future analysis, however, an inductive approach will be maintained to allow new themes to emerge as the data collection continues. Findings will be fed back regularly to the national health service for action and response with the view that the on-going work will aim to inform training and support for contact tracers. It is anticipated that this form of data collection can also operate as a form of debriefing, enabling participants to share their emotional burden and be directed towards established psychological or occupational health support services if required.

### Ethnographic observations

We will conduct ethnographic observations in the contact tracing settings to understand the current processes, practices and challenges experienced by contact tracers as the situation develops and as processes change and adapt to new learning. Ethnography is the study of social interactions, behaviours, and perceptions that occur within a specific environment ^35^. Ethnographic research aims to provide rich, holistic and nuanced insights into people’s views and actions, as well as the nature of the context in which they operate ^35^. Ethnography is an appropriate methodology for studying healthcare practices and processes and can constitute a key step towards the effective design, implementation, and the evaluation of interventions in healthcare.

Two dedicated research staff (ROD and HFMQ) on the project will carry out the observations. Observation sites will be selected based on an effort to collect data from multiple different sites across the country and assuming permission to access from CTC leads and consent of staff. The researchers will meet regularly, and a bespoke observation template will be iteratively developed at the project outset and refined as necessary to ensure consistency in the data being collected by each researcher and across sites. Observations will collect data related to the working context, processes being observed, and challenges being experienced by contact tracers in real time. On observation days, the researchers will ‘shadow’ one site coordinator to capture real time challenges and experiences. Observations will take place up to twice per week in participating contact tracing sites for up to three hours (during the six-month data collection period). Observations will only be conducted if public health guidance allows for this during the period of data collection. No specific, identifiable case/individual data or participant data will be recorded. The objective of observation within contact tracing centres is to understand and collate common concerns, questions, reflect on the impact on staff and to provide insight into the level of peer support/debriefing available following shifts. Consent will be obtained from all contact tracers in the area/room being observed. If any participant does not consent, observations will not be conducted in that area/room. Observation data will be analysed using inductive thematic analysis ^33^.

### Outcomes

Primary quantitative outcomes of this work include the mean self-reported emotional exhaustion ^27^, perceived stress ^28^, anxiety ^30^, posttraumatic stress reactions ^31^ and general health ^29^ of contact tracing staff. Scales will be scored consistent with published guidance and results will be compared across data collection time points with reference to general population and healthcare samples from published research. Primary qualitative outcomes from this work include the experiences of participants engaged in contact tracing as well as systems and process issues, challenges and matters of concern or confusion among the public. 

### Data triangulation

In line with the features of rapid evaluation and appraisal methods ^36^, the multiple data sources collected will be triangulated during analysis. A data triangulation matrix will be used to compare results from across different data collection sources and across different time points ^37, 38^. All data collected around the same time point will be analysed together to assimilate key findings to provide a snapshot of the psychological impact on contact tracers and how well processes and structures are working at that time. Interviews will not be identifiable but will be associated with the contact tracing centre where the contact tracer is working in order to compare experiences across contact tracing centres.

### Dissemination plan

The goal of this research is to quickly provide updates and recommendations to the HSE CMP to support contact tracing staff and the effectiveness of contact tracing in containing the outbreak. We will analyse data on an on-going basis and feedback findings regularly for action and response with the view that the on-going work will aim to inform training and support for contact tracers. This will ensure regular research outputs and updates are fed directly into the CMP team for action. Dissemination will begin as soon as the first round of data is available.

### Ethical considerations

Ethical approval has been obtained from the University College Dublin Ethics Committee (Ref: LS-20-78). Unique (pseudo-anonymous) participant identifiers will be used to link participants’ survey responses over time. It will not be possible to link responses to any individual. Interviews with participants will be anonymised and observations will use pseudonyms rather than identify the site of data collection. It is possible that taking part in the interviews may cause individuals to recall distressing experiences. Consistent with best practice, participants will be advised they are free to refuse to answer questions, are free to withdraw any time without question or reason and are free to take a break during the interview. Participants who share their emotional burden may be directed to further psychological or occupational health support services, if required.

### Study status

We are currently in the middle of our first round of data collection. Once that has finished, we will start the first round of data analysis.

## Discussion

This research project aims to explore contact tracers’ experiences, including the psychological impact of working as a contact tracer during a pandemic, challenges they face, and the information needs and supports they identify. Given the crucial role of contact tracers in managing COVID-19, they can provide useful insights that will support and promote tracing efforts. Regular briefs will be provided to the health service with data, analysis, and recommendations to inform and support the work of contact tracing. Specifically, feedback will be delivered on systems issues, such as IT or contact tracing process issues; challenges experienced by contact tracers; key concerns or questions expressed by positive cases and their contacts; frequently asked questions received by contact tracers from the general public; any questions frequently asked by contact tracers themselves; and the self-reported well-being of contact tracers *.* This research will capture problems as they emerge and rapidly feed this information back to the national health service. Understanding and addressing issues and challenges as they emerge will be critical to improving contact tracing efforts to effectively terminate COVID-19 transmission, but also to inform preventive strategies, training, and response planning for future large-scale infectious disease outbreaks.

A mixed-methods approach will be used to provide a rich understanding of the experiences of contact tracers. Ethnographic observations and interviews will explore the current processes, practices and challenges experienced by contact tracers. This will be an opportunity to provide insight into the level of peer support/debriefing available following shifts and to inform training and support for contact tracers. An online survey will measure participants self-reported levels of stress, anxiety, emotional exhaustion, and general health. The survey will also include some open text responses to capture specific/common issues raised during calls with cases/contacts. The analysis will inform the improvement of contact tracing efforts to effectively terminate COVID-19 transmission, as well as preventive strategies and response planning for future large-scale infectious disease outbreaks.

This study will offer much needed insight into the psychological impact of working in contact tracing. The complexity and sensitive nature of contact tracing has been identified ^15^, and there is a clear need for research to understand the psychological impact of this work and how they may be best supported in the crucial role of contact tracing. Collaborating closely with the HSE CMP will facilitate the rapid implementation of findings to inform training and support for contact tracers and enhancement of contact tracing processes.

## Data availability

The data referenced by this article are under copyright with the following copyright statement: Copyright: ï¿½ 2021 O'Donovan R et al.

Data associated with the article are available under the terms of the Creative Commons Attribution Licence, which permits unrestricted use, distribution, and reproduction in any medium, provided the original data is properly cited. 

### Underlying data

No data are associated with this article.

### Extended data

Research Repository UCD: Contact tracing during the COVID-19 outbreak: a protocol for enabling rapid learning from experiences and exploring the psychological impact on contact tracers. Reference Source ^26^.

This project contains the following extended data:

- Data collection materials

Data are available under the terms of the Creative Commons Attribution 3.0 Ireland license (CC-BY 4.0).
