# Supplementary material for: Contact tracing during the COVID-19 outbreak: a protocol for enabling rapid learning from experiences and exploring the psychological impact on contact tracers
Source: HRB Open Res. 2021 Sep 15;4:33. Originally published 2021 Mar 30. [Version 2] doi: 10.12688/hrbopenres.13236.2 (PMC8485097; doi:10.12688/hrbopenres.13236.2)
Supplement: Peer review history

# Peer review: Reviewer response for version 2

**DOI:** 10.21956/hrbopenres.14594.r30308

**Published:** 2021-09-30

**Authors:** Anne Marie Liebel

**Affiliations:** 1 Health Communication Partners LLC, Simpsonville, SC, USA

The team has made such careful and care-filled revision to an already strong paper, about a timely and important project. Bravo!

Is the study design appropriate for the research question?

Partly

Is the rationale for, and objectives of, the study clearly described?

Yes

Are sufficient details of the methods provided to allow replication by others?

Partly

Are the datasets clearly presented in a useable and accessible format?

Not applicable

Reviewer Expertise:

Professional learning; teaching and learning

I confirm that I have read this submission and believe that I have an appropriate level of expertise to confirm that it is of an acceptable scientific standard.

# Peer review: Reviewer response for version 2

**DOI:** 10.21956/hrbopenres.14594.r30307

**Published:** 2021-09-27

**Authors:** Robert ffrench-O'Carroll, Eoin O'Rathallaigh

**Affiliations:** 1 Department of Anesthesiology, BC Women's Hospital Vancouver, Vancouver, Canada; 2 Department of Anaesthesiology, Cork University Hospital, Cork, Ireland

Dear authors, 

 Thank you for considering our recommendations and revising your protocol. We are satisfied that you have addressed all of our concerns and we wish you all the best with this important study. 

 Regards

 Dr Robert ffrench-O'Carroll & Dr Eoin O'Rathallaigh

Is the study design appropriate for the research question?

Yes

Is the rationale for, and objectives of, the study clearly described?

Yes

Are sufficient details of the methods provided to allow replication by others?

Partly

Are the datasets clearly presented in a useable and accessible format?

Not applicable

Reviewer Expertise:

Healthcare worker well-being, psychological impact of COVID-19 on healthcare staff, Survey studies.

We confirm that we have read this submission and believe that we have an appropriate level of expertise to confirm that it is of an acceptable scientific standard.

# Peer review: Reviewer response for version 1

**DOI:** 10.21956/hrbopenres.14398.r29675

**Published:** 2021-07-29

**Authors:** Robert ffrench-O'Carroll, Eoin O'Rathallaigh

**Affiliations:** 1 Department of Anesthesiology, BC Women's Hospital Vancouver, Vancouver, Canada; 2 Department of Anaesthesiology, Cork University Hospital, Cork, Ireland

The authors provide their protocol for a study exploring the challenges experienced by contact tracers during the COVID-19 outbreak along with the psychological impact of their work. We are impressed by several elements of this protocol.

 Firstly, the authors clearly outline the rationale for conducting such a study. Contact tracers are pivotal to control of COVID-19 yet the challenges of the work they do and its potential impact on their psychological well-being is under explored in the literature. The authors are to be commended for their mixed-methods approach, use of data triangulation and their aim to provide regular feedback of results. They are to be commended for their use of validated tools to assess psychological well-being and their use of interviews and ethnographic observations, which will no doubt capture lots of rich qualitative data. Finally there has been lots of research around psychological stress of COVID-19 on healthcare workers. What makes this study particularly interesting is the the focus on contact tracers who come from varied backgrounds and may face unique challenges. 

 We have several minor reservations and recommendations, which the authors may wish to address at this stage in their research; 

 Firstly the aims of the study are broad. Authors wish to assess both systems problems and challenges with contact tracing along with the psychological impact of the work. Do the authors wish to explore the 'current processes, practices and challenges experienced by contact tracers' in order to paint a better picture of the factors impacting their well-being? or are the authors just interested in the work processes? if it's the latter, then the two research aims may be better explored by separate research studies as they are quite distinct aims (although likely related). 

 Regarding the administration of the questionnaire, why the three time points two months apart? how was this time frame decided? The authors should elaborate on this. Also the distress will no doubt vary with the stage of the pandemic (at the beginning/during a wave); the authors should comment on this during their protocol. 

 The authors should comment on the expected response rate of their questionnaire and what response rate they are aiming for.

 What factors/responses/observed behaviours or reported measures of psychological distress would prompt direction to further supports?

 ' *Participants who share their emotional burden may be directed to further psychological or occupational health support services, if required.' *What factors/responses/observed behaviours or reported measures of psychological distress would prompt direction to further supports?

 The authors should comment on how long the questionnaire takes to complete and any methods they will use to ensure they achieve their desired response rate 

 How regular will feedback be provided regarding study results?

 The authors mention they will explore supports used by contact tracers. Will this be only covered in interviews? they may consider capturing more data on this using open ended questions on the survey. Also many healthcare workers don't use supports that are available to them; it would be interesting to explore some of the barriers to accessing supports for contact tracers. Also do contact tracers need more tailored supports compared to other workers? 

 In the discussion section we suggest changing “contact tracing efforts to effectively terminate COVID-19 transmission” to “improving the efficacy of contact tracing systems during the COVID-19 pandemic”. We are suggesting this as as the discussion may be biased towards the work of contact tracing in management of SARS-CoV-2 transmission. Our change aims to respect and acknowledge their role, but we are mindful that their role is part of a coordinated effort at all levels – public, government, administrative and frontline healthcare.

Is the study design appropriate for the research question?

Yes

Is the rationale for, and objectives of, the study clearly described?

Yes

Are sufficient details of the methods provided to allow replication by others?

Partly

Are the datasets clearly presented in a useable and accessible format?

Not applicable

Reviewer Expertise:

Healthcare worker well-being, psychological impact of COVID-19 on healthcare staff, Survey studies.

We confirm that we have read this submission and believe that we have an appropriate level of expertise to confirm that it is of an acceptable scientific standard, however we have significant reservations, as outlined above.

# Response

**Published:** 2021-08-16

**Authors:** Róisín Róisín

**Affiliations:** University College Dublin, Ireland

Thank you for your review and valuable suggestions. Please find below our response to your comments and an account of the changes that have been made to the manuscript.

*Firstly, the authors clearly outline the rationale for conducting such a study. Contact tracers are pivotal to control of COVID-19 yet the challenges of the work they do and its potential impact on their psychological well-being is under explored in the literature. The authors are to be commended for their mixed-methods approach, use of data triangulation and their aim to provide regular feedback of results. They are to be commended for their use of validated tools to assess psychological well-being and their use of interviews and ethnographic observations, which will no doubt capture lots of rich qualitative data. Finally there has been lots of research around psychological stress of COVID-19 on healthcare workers. What makes this study particularly interesting is the focus on contact tracers who come from varied backgrounds and may face unique challenges.*

 Thank you for taking the time to review our protocol and for outlining these key strengths of this research.

*We have several minor reservations and recommendations, which the authors may wish to address at this stage in their research;*

*Firstly the aims of the study are broad. Authors wish to assess both systems problems and challenges with contact tracing along with the psychological impact of the work. Do the authors wish to explore the 'current processes, practices and challenges experienced by contact tracers' in order to paint a better picture of the factors impacting their well-being? or are the authors just interested in the work processes? if it's the latter, then the two research aims may be better explored by separate research studies as they are quite distinct aims (although likely related).*

 We wish to address both of these aims in our research: to explore the ways current processes, practices and challenges experienced by contact tracers’ impact both their well-being and the efficiency of the work processes they undertake, as we contend these are likely closely related. We will explore this broad aim through surveys and interviews. Interviews and observations will also provide insight into contact tracers work processes and they ways in which these influence their experiences. We aim to explore both of these issues together to provide a better understanding of contact tracers experiences.

*Regarding the administration of the questionnaire, why the three time points two months apart? how was this time frame decided? The authors should elaborate on this. Also the distress will no doubt vary with the stage of the pandemic (at the beginning/during a wave); the authors should comment on this during their protocol.*

 We have now included the likelihood that contact tracers’ experiences will likely vary at different stages of the pandemic as a rationale for our longitudinal approach. The two months between data collection time points will provide time for analysis prior to the next time point, allowing for additional probes to be added to the interview guide, if required.

*The authors should comment on the expected response rate of their questionnaire and what response rate they are aiming for.*

 We have now included a target response rate.

*'Participants who share their emotional burden may be directed to further psychological or occupational health support services, if required.' What factors/responses/observed behaviours or reported measures of psychological distress would prompt direction to further supports?*

 We have now clarified that participants who display or report significant emotional distress during interviews or who report high levels of emotional distress in survey measures may be directed to further psychological or occupational health support services, if required.

*The authors should comment on how long the questionnaire takes to complete and any methods they will use to ensure they achieve their desired response rate*

 We have now clarified that the survey takes 8-10 minutes to complete and that reminders to complete the survey will be sent to contact tracing centres during each data collection period.

*How regular will feedback be provided regarding study results?*

 Feedback will be provided following each round of data collection. We have now clarified this in our dissemination sections.

*The authors mention they will explore supports used by contact tracers. Will this be only covered in interviews? they may consider capturing more data on this using open ended questions on the survey. Also many healthcare workers don't use supports that are available to them; it would be interesting to explore some of the barriers to accessing supports for contact tracers. Also do contact tracers need more tailored supports compared to other workers?*

 We will explore the supports used by contact tracers in each form of data collection. Interviews will cover the supports that contact tracers are aware of and avail of, as well as the barriers to accessing the available supports and perceptions that there is a lack of support available. Sources of support and communication around the available supports will be recorded during ethnographic observations. We have now clarified this in our methodology section.

*In the discussion section we suggest changing “contact tracing efforts to effectively terminate COVID-19 transmission” to “improving the efficacy of contact tracing systems during the COVID-19 pandemic”. We are suggesting this as as the discussion may be biased towards the work of contact tracing in management of SARS-CoV-2 transmission. Our change aims to respect and acknowledge their role, but we are mindful that their role is part of a coordinated effort at all levels – public, government, administrative and frontline healthcare.*

 Thank you for this suggestion. We have revised this line in our discussion section accordingly.

# Peer review: Reviewer response for version 1

**DOI:** 10.21956/hrbopenres.14398.r29233

**Published:** 2021-04-19

**Authors:** Anne Marie Liebel

**Affiliations:** 1 Health Communication Partners LLC, Simpsonville, SC, USA

Thank you for the opportunity to review this study protocol for the support of contact tracers in the Irish health system. The rationale and objective were clearly described and certainly of urgent importance. Data collection processes are well described, along with ethical concerns, and issues of confidentiality and triangulation. Mixed methods seem appropriate and without undue burden on participants.

 I agree enthusiastically that “There is considerable potential to harness the experience of contact tracers in the pivotal role they play by identifying needs and issues as they emerge in this rapidly changing situation. This includes challenges experienced during the contact tracing process as well as patterns in the public’s understanding of, and reported adherence to, the public health measures.”

 I believe the authors likely have answers for all my queries below, so it is not a matter of any flaws in the design but instead articulating some assumptions and sharing more detail about the study as they imagine and intend it. 

 Figure 1 “The overview of the study design” seems instead to be an overview of data collection processes, with a mention of data analysis.

 More detail of why particular data will be collected, and how data will be analyzed, would help reviewers and guide replication. For example, it is unclear what will be done with interview data “in-depth responses about the contact tracers’ experiences and challenges.” How will this be analyzed, how will insights be gleaned, and what will be done with them?

 What are we to understand the relationship is between this data and the “rapid learning from experience” in the title? Who is understood to be learning, and from whom?

 It is yet unclear what will be done with the resulting dataset beyond “provide updates and recommendations to the HSE CMP to support contact tracing staff and the effectiveness of contact tracing in containing the outbreak.”

 For example, what kinds of analysis are expected to result in usable information? In what form will data be shared? What is assumed about the usability of the information to the HSE CMP? How will the data “inform training and support for contact tracers” and move toward fulfilling the promise in the study title? I certainly hope it will, as this is a real (potential) strength of the study!

Is the study design appropriate for the research question?

Partly

Is the rationale for, and objectives of, the study clearly described?

Yes

Are sufficient details of the methods provided to allow replication by others?

Partly

Are the datasets clearly presented in a useable and accessible format?

Not applicable

Reviewer Expertise:

Professional learning; teaching and learning

I confirm that I have read this submission and believe that I have an appropriate level of expertise to confirm that it is of an acceptable scientific standard, however I have significant reservations, as outlined above.

# Response

**Published:** 2021-08-16

**Authors:** Róisín Róisín

**Affiliations:** University College Dublin, Ireland

Thank your for taking the time to review of our manuscript. We are very grateful for your valuable suggestions for improvements. Please find below our response to your comments and an account of the changes that have been made to the manuscript.

*Figure 1 “The overview of the study design” seems instead to be an overview of data collection processes, with a mention of data analysis.*

 Thank you, we have now re-labelled Figure 1 as “overview of data collection” and we now provide further details regarding data analysis within the methods section.

*More detail of why particular data will be collected, and how data will be analyzed, would help reviewers and guide replication. For example, it is unclear what will be done with interview data “in-depth responses about the contact tracers’ experiences and challenges.” How will this be analyzed, how will insights be gleaned, and what will be done with them?*

 We have now included further detail regarding the rationale for each data collection source. Our revised manuscript also provides further description of data analysis and our plans for dissemination of findings and implementation of recommendations.

*What are we to understand the relationship is between this data and the “rapid learning from experience” in the title? Who is understood to be learning, and from whom?*

 The rapid learning related to the ongoing and regular feedback being provided to the COVID-19 Contact Management Programme (CMP). This feedback will be provided at the end of each data collection point in the form of evidence briefs and presentations. They will include survey results and emerging themes from the interviews, with contextual, real-time information being provided from the ethnographic observation.

*It is yet unclear what will be done with the resulting dataset beyond “provide updates and recommendations to the HSE CMP to support contact tracing staff and the effectiveness of contact tracing in containing the outbreak.” For example, what kinds of analysis are expected to result in usable information? In what form will data be shared? What is assumed about the usability of the information to the HSE CMP? How will the data “inform training and support for contact tracers” and move toward fulfilling the promise in the study title? I certainly hope it will, as this is a real (potential) strength of the study!*

 Thank you. We have now expanded our dissemination plan to address these issues and clarify how analysis will be used to inform future training and support for contact tracers. Specially, our evidence briefs submitted to the CMP at each time point will include recommendations based on our findings, where appropriate. Given the links between the research team and our collaborators within the CMP, we hope this will constitute an avenue to directly inform and influence implementation and policy.
